# Major and minor perineural invasion in salivary gland cancer

**DOI:** 10.3389/fonc.2024.1466196

**Published:** 2025-01-17

**Authors:** Fei Liu, Yinglin Chu, Qizhe Zheng, Yunshuang Hu, Yiyi Wang, Lu Qin, Shuaikun Fu, Suping Wang

**Affiliations:** ^1^ Department of Oral Medicine, The First Affiliated Hospital of Zhengzhou University, Zhengzhou, China; ^2^ Department of Oral Medicine, The First Affiliated Hospital of Xinxiang Medical College, Xinxiang, China

**Keywords:** salivary gland cancer, perineural invasion, adjuvant radiotherapy, chemoradiation, survival

## Abstract

**Objective:**

To delineate the distribution of perineural invasion (PNI), evaluate its impact on patient survival, and identify optimal criteria for initiating adjuvant radiation therapy (RT) in cases of PNI associated with salivary gland cancer (SGC).

**Methods:**

This retrospective study categorized enrolled patients into three groups based on PNI status (none, minor, or major), defined by the extent of nerve involvement. The influence of PNI on overall survival and locoregional control was assessed using a Cox proportional hazards model.

**Results:**

A total of 555 patients were incorporated into the study. Logistic regression analysis indicated that tumor stage, neck stage, histological grade, and pathological type were independently linked to the occurrence of PNI. In the Cox model assessing overall survival, patients exhibiting minor nerve PNI demonstrated a hazard ratio (HR) of 1.78 [95% CI: 1.14-2.47] in comparison to those without PNI, a difference that was statistically significant (p<0.001). Conversely, the variation in HR between patients with major nerve PNI and those with minor nerve PNI was not statistically significant (p=0.673). In the Cox model for locoregional control, patients with minor and major nerve PNI exhibited HRs of 1.64 [95% CI: 1.17-2.78] and 1.65 [95% CI: 1.03-2.90], respectively, when compared to those without PNI. Subgroup analyses revealed that the incorporation of chemotherapy into radiotherapy did not significantly modify the risk of mortality or locoregional recurrence in comparison to patients treated with radiotherapy alone, irrespective of PNI classification.

**Conclusion:**

Both minor and major nerve PNI exerting comparable influences on prognosis, the adjunctive use of chemotherapy in combination with RT did not yield improvements in overall survival or locoregional control, irrespective of PNI status.

## Introduction

Salivary gland cancer (SGC), while relatively uncommon, comprises less than 5% of all head and neck malignancies (1). The primary treatment of choice typically involves complete excision of the primary site, with or without neck dissection. Adjuvant therapy becomes necessary in the presence of adverse pathological features, with perineural invasion (PNI) standing out as a crucial prognostic factor (2). As per the NCCN guidelines, the presence of PNI indicates a poorer prognosis, necessitating adjuvant radiotherapy (RT) to enhance locoregional control (3).

Owing to the intricate network of small (non-named) and large (named) nerves in the head and neck region, the invasive patterns of PNI can vary (4). While this phenomenon has been acknowledged in the past, it has been subject to limited analysis. Minor nerve involvement prevails over major nerve involvement in laryngeal and hypopharyngeal cancers, correlating with a heightened risk of local recurrence (5). In the case of salivary gland mucoepidermoid carcinoma (MEC), significant mortality prediction is linked to large nerve involvement as opposed to small nerve involvement (6). These studies shed light on the distinct distribution and survival implications between major and minor nerve PNI; however, considerable gaps persist, including the comparison between no PNI and minor nerve PNI, as well as the identification of the PNI type indicating the necessity of RT.

Hence, our objective was to chart the distribution of PNI, ascertain its survival implications, and delineate the optimal indicator for initiating RT in PNI cases associated with SGC.

## Patients and methods

### Ethical approval

This study was approved by Zhengzhou University Institutional Research Committee, and written informed consent for medical research was obtained from all patients before starting the treatment. All methods were performed in accordance with the relevant guidelines and regulations.

### Study design

In order to fulfill our objectives, a retrospective investigation was undertaken. Records of patients (>18 years) who underwent surgical treatment for primary SGC were scrutinized from January 2000 to May 2019. Inclusion criteria stipulated: availability of PNI data, accessible pathologic sections for reevaluation, and obtainment of comprehensive follow-up details (≥ 5 years). Patients with a history of previous malignancies were excluded. Pertinent information regarding demographics, pathology, treatment modalities, and post-treatment monitoring was extracted.

### Study variables

Tumor and neck staging were determined in accordance with the 8^th^ edition of the AJCC classification. Pathological sections were meticulously reviewed by a minimum of two head and neck pathologists. Histological grade was stratified as low, intermediate, or high based on the 5th edition of the World Health Organization Classification for salivary gland tumors (7). The presence of lymphovascular invasion (LVI) was ascertained if cancer cells were detected within lymphatic vessels. PNI was verified if cancer cells invaded a nerve, categorized as major when involving facial nerves, the hypoglossal ansa, spinal nerves, hypoglossal nerves, branches of the cervical plexus, and other major nerves, or minor.

The primary outcome variables were the 5-year overall survival (OS) and 5-year locoregional control (LRC). OS duration was computed from the surgical date to the date of death or last follow-up, while LRC time spanned from surgery to the initial locoregional recurrence or last follow-up. The secondary outcome variables included the distribution and predictive factors associated with minor and major nerve PNI.

### Treatment

Each patient underwent a comprehensive evaluation involving ultrasound and CT scans as baseline assessments, with intermittent utilization of PET-CT scans for evaluating cervical and distant metastases. Frozen sections of the primary tumor and margins were consistently scrutinized during surgeries to ensure accurate pathological assessments. Neck dissection was conducted when positive lymph nodes were detected clinically or pathologically. For non-parotid gland tumors, neck dissections encompassed levels I to III, while parotid gland cancers involved levels I to III and Va. In cases where high-risk factors were identified, adjuvant RT formed part of the therapeutic strategy. The presence of extranodal extension or positive margins prompted consideration of adjuvant chemotherapy (CRT).

### Statistical analysis

The data deficiency within variables of tumor stage, pathologic grade, PNI, and LVI displayed a non-random missing pattern (8), with missing data rates ranging from 15.5% to 19.2%. To mitigate this, a meticulous imputation process was undertaken using the Multiple Imputation by Chained Equations algorithm with Fully Conditional Specifications (9).

For the primary outcome variable, the impact of none, minor, and major nerve PNI on OS and LRC was explored via univariate Cox models. Subsequently, significant factors were assessed through a multivariable Cox model, with outcomes presented as hazard ratios (HR) and 95% confidence intervals (CI). Additionally, the potential enhancement of prognosis by adjuvant chemotherapy in conjunction with RT was evaluated based on differing PNI statuses.

Regarding the secondary outcome variable, patients were categorized into PNI status groups for comparison through the Chi-square test for clinicopathologic factors. Subsequently, significant factors were scrutinized using logistic regression, with results illustrated as odds ratios (OR) and 95% CIs. All statistical analyses were executed utilizing R 3.4.4, with a significance level set at p < 0.05.

## Results

### Baseline data

A total of 555 patients were included in the study, comprising 217 males and 338 females, with a mean age of 50 ± 17 years. The primary sites were predominantly major glands in 400 patients (72.1%) and minor glands in 155 patients (27.9%). The majority of patients (n=338, 60.9%) presented with T3/4 tumors, and lymph node metastasis was observed in 146 patients (26.3%). Histologic grading revealed low grade in 280 patients (50.5%), intermediate grade in 166 patients (29.9%), and high grade in 109 patients (19.6%). Mucoepidermoid carcinoma was the most prevalent pathologic type (n=194, 35.0%), followed by adenoid cystic carcinoma (n=175, 51.5%). LVI was identified in 127 patients (22.9%). Positive margin occurred in 52 (9.4%) patients. In total, 233 (42.0%) patients received RT, in whom 99 cases also received adjuvant chemotherapy. During our follow-up with a median time of 5 years, there were 170 locoregional recurrences and 200 deaths.

### Predictor for PNI

PNI was documented in 146 patients (26.3%), among whom 100 exhibited minor nerve PNI and 46 had major nerve PNI. The three subgroups exhibited notable distinctions in terms of tumor stage, neck stage, histologic grade, pathologic type, and LVI. Patients with major nerve PNI tended to have T3/4 tumors, high-grade histology, lymph node metastasis, LVI, and a diagnosis of adenoid cystic carcinoma (**Table 1**). These factors were further evaluated in multivariable analysis.

**Table 1:** Baseline data of patients with salivary gland cancer.

Variable	Total	Perineural invasion (n=146)			p*
		None (n=409)	Minor (n=100)	Major (n=46)	
Age					
emsp≤50	269 (48.4%)	199 (48.7%)	50 (50.0%)	20 (43.5%)	0.756
>50	286 (51.6%)	210 (51.3%)	50 (50.0%)	26 (56.5%)	
Sex					
Male	217 (39.1%)	163 (39.9%)	35 (35.0%)	19 (41.3%)	0.638
Female	338 (60.9%)	246 (60.1%)	65 (65.0%)	27 (58.7%)	
Involved gland					
Major	400 (72.1%)	299 (73.1%)	70 (70.0%)	31 (67.4%)	0.628
Minor	155 (27.9%)	110 (26.9%)	30 (30.0%)	15 (32.6%)	
Tumor stage					
T1/2	338 (60.9%)	279 (68.2%)	50 (50.0%)	9 (19.6%)	<0.001
T3/4	217 (39.1%)	130 (31.8%)	50 (50.0%)	37 (80.4%)	
Neck stage					
N0	409 (73.7%)	330 (80.7%)	63 (63.0%)	16 (34.8%)	<0.001
N+	146 (26.3%)	79 (19.3%)	37 (37.0%)	30 (65.2%)	
Grade					
Low	280 (50.5%)	254 (62.1%)	18 (18.0%)	8 (17.4%)	<0.001
Intermediate	166 (29.9%)	102 (24.9%)	50 (50.0%)	14 (30.4%)	
High	109 (19.6%)	53 (13.0%)	32 (32.0%)	24 (52.2%)	
Type^&^					
MEC	194 (35.0%)	166 (40.6%)	20 (20.0%)	8 (17.4%)	<0.001
ACC	175 (31.5%)	99 (22.0%)	46 (46.0%)	30 (65.2%)	
Others	186 (33.5%)	144 (35.2%)	34 (34.0%)	8 (17.4%)	
LVI^					
No	428 (77.1%)	374 (92.4%)	40 (40.0%)	14 (30.4%)	<0.001
Yes	127 (22.9%)	35 (7.6%)	60 (60.0%)	32 (69.6%)	

^*^ Comparison in patients with different perineural invasion status.

^&^ MEC, mucoepidermoid carcinoma; ACC, adenoid cystic carcinoma.

^^^ LVI, lymphovascular invasion.

The logistic regression analysis revealed that tumor stage, neck stage, histologic grade, and pathologic type were independently associated with the occurrence of PNI. In comparison to T1/2 stage, T3/4 stage was associated with an OR of 2.18 [95%CI: 1.34-3.81]. The presence of lymph node metastasis was linked to a higher likelihood of PNI, with an OR of 1.87 [95%CI: 1.26-3.76]. While low and intermediate grades exhibited comparable probabilities of PNI (p=0.135), high grade was associated with an OR of 2.43 [95%CI: 1.29-4.55]. Among the pathologic types, adenoid cystic carcinoma demonstrated the highest incidence of PNI occurrence, while LVI did not appear to impact the development of PNI (**Table 2**).

**Table 2:** Logistic regression of predictors for perineural invasion.

Variable	p	Odds ratio [95% confidence interval]
Tumor stage		
T1/2 (n=338)		ref
T3/4 (n=217)	<0.001	2.18 [1.34-3.81]
Neck stage		
N0 (n=409)		ref
N+ (n=146)	<0.001	1.87 [1.26-3.76]
Grade		
Low (n=280)		ref
Intermediate (n=166)	0.135	1.34 [0.77-1.99]
High (n=109)	<0.001	2.43 [1.29-4.55]
Type&		
ACC (n=175)		ref
Others (n=186)	<0.001	0.88 [0.72-0.95]
MEC (n=194)	<0.001	0.90 [0.84-0.96]
LVI^		
No (n=428)		ref
Yes (n=127)	0.367	1.46 [0.87-2.00]

& MEC, mucoepidermoid carcinoma; ACC, adenoid cystic carcinoma.

^ LVI, lymphovascular invasion.

### Univariate analysis

Factors such as tumor stage, neck stage, histologic grade, pathologic type, LVI, PNI, adjuvant therapy, and margin status were found to be associated with both OS and LRC (all p<0.005, **Table 3**) in the univariate analysis. These variables were further evaluated in a multivariable Cox model. Age and sex, however, did not exhibit significant associations with OS or LRC.

**Table 3:** Predictors for overall survival and locoregional control.

Variable	Overall survival				Locoregional control			
	Univariate		Multivariate		Univariate		Multivariate	
	p	HR [95%CI]	p	HR [95%CI]	p	HR [95%CI]	p	HR [95%CI]
Age								
≤50 (n=269)		ref				ref		
>50 (n=286)	0.538	1.45 [0.56-1.98]			0.279	1.56 [0.34-1.97]		
Sex								
Male (n=217)		ref				ref		
Female (n=338)	0.732	1.37 [0.64-2.03]			0.556	1.41 [0.52-1.97]		
Involved gland								
Major (n=400)		ref		ref		ref		ref
Minor (n=155)	<0.001	1.63 [1.21-2.41]	0.164	1.54 [0.93-2.11]	<0.001	1.55 [1.17-2.50]	0.276	1.42 [0.91-2.02]
Tumor stage								
T1/2 (n=338)		ref		ref		ref		ref
T3/4 (n=217)	<0.001	2.19 [1.39-3.00]	<0.001	2.13 [1.25-3.82]	<0.001	2.31 [1.18-3.78]	<0.001	2.17 [1.18-3.43]
Neck stage								
N0 (n=409)		ref		ref		ref		ref
N+ (n=146 )	<0.001	1.73 [1.11-2.85]	<0.001	1.57 [1.11-1.90]	<0.001	1.61 [1.18-2.90]	<0.001	1.56 [1.13-1.84]
Grade								
Low (n=280)		ref		ref		ref		ref
Intermediate (n=166)	0.102	1.23 [0.83-2.11]	0.065	1.45 [0.94-2.06]	0.213	1.42 [0.63-2.36]	0.087	1.20 [0.88-2.32]
High (n=109)	<0.001	2.78 [1.43-5.39]	<0.001	2.44 [1.18-3.75]	<0.001	2.78 [1.43-5.39]	<0.001	2.15 [1.22-3.61]
Type&								
ACC (n=175)		ref		ref		ref		ref
MEC (n=194)	0.227	1.11 [0.68-1.88]	0.134	1.16 [0.73-2.65]	0.306	1.21 [0.54-2.19]	0.264	1.20 [0.64-2.89]
Others (n=186)	<0.001	2.01 [1.10-3.27]	0.227	1.20 [0.65-2.80]	<0.001	2.01 [1.10-3.27]	0.389	1.45 [0.83-4.54]
LVI^								
No (n=428)		ref		ref		ref		ref
Yes (n=127)	<0.001	1.67 [1.03-1.98]	0.190	1.42 [0.68-2.35]	<0.001	1.54 [1.10-2.00]	0.274	1.37 [0.70-2.42]
PNI~								
No (n=409)		ref		ref		ref		ref
Minor (n=100)	<0.001	1.83 [1.20-2.67]	<0.001	1.78 [1.14-2.47]	<0.001	1.67 [1.13-2.78]	<0.001	1.64 [1.17-2.78]
Major (n=46)	<0.001	1.85 [1.24-3.00]	<0.001	1.81 [1.19-2.99]	<0.001	1.81 [1.09-3.45]	<0.001	1.65 [1.03-2.90]
Adjuvant therapy@								
None (n=322)		ref		ref		ref		ref
RT (n=134)	<0.001	0.92 [0.65-0.99]	<0.001	0.87 [0.74-0.95]	<0.001	0.91 [0.68-0.99]	<0.001	0.88 [0.72-0.96]
CRT (n=99)	<0.001	0.90 [0.74-0.99]	<0.001	0.88 [0.70-0.97]	<0.001	0.89 [0.64-0.99]	<0.001	0.87 [0.69-0.95]
Margin								
No (n=503)		ref		ref		ref		
Yes (n=52)	<0.001	2.45 [1.20-4.12]	<0.001	2.28 [1.17-3.86]	<0.001	2.58 [1.19-4.75]	<0.001	2.15 [1.30-3.57]

& MEC, mucoepidermoid carcinoma; ACC, adenoid cystic carcinoma.

^ LVI, lymphovascular invasion.

~ PNI, perineural invasion.

@ CRT, chemoradiation; RT, radiation.

### Multivariable analysis

In the Cox model for OS, patients with no PNI had a hazard ratio (HR) of 1.78 [95%CI: 1.14-2.47], compared to those with minor nerve PNI, and this discrepancy was statistically significant (p<0.001). Conversely, the difference in HR between patients with major nerve PNI and those with minor nerve PNI was not significant (p=0.673). Additionally, other independent factors affecting OS included tumor stage, neck stage, histologic grade, adjuvant therapy, and margin status (**Table 3**).

In the Cox model for LRC, patients with minor and major nerve PNI exhibited HRs of 1.64 [95% CI: 1.17-2.78] and 1.65 [95% CI: 1.03-2.90], respectively, when compared to those without PNI. Similar to OS, independent factors influencing LRC comprised tumor stage, neck stage, histologic grade, adjuvant therapy, and margin status (**Table 3**).

In a further analysis assessing whether the impact of PNI status on OS and LRC was influenced by the primary site, it was observed that patients with major nerve PNI exhibited comparable HRs to those with minor nerve PNI. However, the cohort without PNI demonstrated significantly reduced HRs, regardless of the primary sites (**Supplementary Table S1**).

### Subgroup analysis

In order to elucidate the impact of adjuvant therapy on OS and LRC stratified by PNI status, a subgroup analysis was conducted. Regarding OS, the addition of chemotherapy to RT did not significantly alter mortality risk compared to patients treated with RT alone, regardless of PNI classification. However, among patients with no or minor nerve PNI, RT led to superior survival compared to no adjuvant therapy, whereas in patients with major nerve PNI, those treated with RT or none exhibited comparable OS (**Table 4**).

**Table 4:** Impact of adjuvant therapy on overall survival and locoregional control stratified by different perineural invasion (PNI) status.

Stratification	Overall survival		Locoregional control	
	p	HR [95%CI]	p	HR [95%CI]
No PNI (n=409)				
RT* (n=84)		ref		ref
CRT (n=39)	0.643	1.10 [0.67-2.05]	0.526	1.18 [0.70-2.03]
No adjuvant therapy (n=286)	<0.001	2.19 [1.36-3.26]	<0.001	2.26 [1.28-3.53]
Minor nerve PNI (n=100)				
RT (n=33)		ref		ref
CRT (n=40)	0.543	1.98 [0.62-4.87]	0.683	1.64 [0.78-3.64]
No adjuvant therapy (n=27)	0.012	4.16 [1.54-8.16]	0.019	3.32 [1.36-6.53]
Major nerve PNI (n=46)				
RT (n=17)		ref		ref
CRT (n=20)	0.943	2.00 [0.54-5.39]	0.888	1.74 [0.63-4.58]
No adjuvant therapy (n=9)	0.148	5.89 [0.67-13.86]	0.046	4.65 [2.20-9.57]

Concerning LRC, the addition of chemotherapy to RT did not improve disease control in comparison to patients treated with RT alone, irrespective of PNI classification. Furthermore, RT provided superior disease control compared to no adjuvant therapy, regardless of PNI classification (**Table 4**).

## Discussion

Our study revealed that PNI occurrence was intricately associated with advanced stage, lymph node metastasis, high histologic grade, and the pathologic type of adenoid cystic carcinoma. Interestingly, both minor and major nerve PNI exerted similar influences on OS and LRC. Moreover, the addition of chemotherapy to RT did not confer improvements in OS or LRC, regardless of PNI status. However, it was notable that RT generally yielded a more favorable prognosis compared to no adjuvant therapy, except in patients with major nerve PNI. This study represents the first of its kind to comprehensively analyze the distribution of PNI and elucidate its significant implications for patient survival. Our results have the potential to inform and guide clinical decision-making regarding adjuvant therapy, thereby offering valuable insights for the management of patients in this context.

PNI is typically considered an adverse pathologic characteristic, and it is relatively prevalent in head and neck cancer, with more than 40% of patients with SGC being affected. However, the debate over its impact on survival persists. For instance, Katabi et al. reported PNI in about 20% of their 72 SGC patients, but found similar OS, disease-specific survival, and recurrence-free survival between those with and without PNI (11). On the other hand, Huyett et al. observed PNI in 46.2% of tumors, and noted that malignancies with PNI were more likely to exhibit advanced T and N classification, high-risk pathologic types, positive margins, and angiolymphatic invasion. Their study also showed that PNI positivity was linked to worse overall and disease-free survival, although this effect became statistically insignificant when controlling for other prognostic factors, age, and adjuvant therapy (4). In contrast, de Melo et al. studied 32 SGC patients and found that PNI-positive tumors had significantly higher risks of developing soft tissue invasion and positive surgical margins, as well as poorer disease-specific survival. They emphasized that PNI-positive SGC is an independent prognostic factor associated with unfavorable outcomes, increased locoregional recurrence, and a more aggressive disease pattern (12). A recent meta-analysis that included 14 studies revealed that patients with PNI had significantly higher rates of mortality, time to recurrence, disease-specific mortality, and distant metastasis when compared to those without PNI (13). The negative predictive value of PNI in prognosis has also been corroborated by our own research and others (10, 14–16). However, variations among these studies may stem from differences in sample size and the accuracy of PNI definition. Of particular significance is our distinction between minor and major nerve PNI in SGC, a phenomenon that has not been previously identified (5). This differentiation is notable for several reasons. First, major nerve PNI was thought to be more likely in major salivary gland cancer due to the course pathway of the facial, lingual, and hypoglossal nerves. However, our analysis did not confirm this deduction, possibly due to the abundant branches of the trigeminal nerve in the oral cavity. Second, major nerve PNI was found to be more common in patients with T3/4 tumors, lymph node metastasis, and high-grade tumors, suggesting that major nerve PNI tends to develop from minor nerve PNI as the disease progresses. Furthermore, it is worth noting the comparable impact of major and minor nerve PNI on OS and LRC. This parallel influence may be explained by the fact that efforts were made to achieve negative margins in all patients during surgery, with the assistance of frozen section analysis. This is underscored by our low rate of positive margins.

Adjuvant therapy plays a pivotal role in the comprehensive management of head and neck cancer, mitigating the risk of both locoregional and distant metastasis. Current clinical guidelines for CRT are predominantly informed by two seminal trials and their subsequent meta-analyses (17–19). The EORTC 22931 trial (17), which enrolled 167 patients with resected stage III/IV head and neck cancer, and the subsequent RTOG trial of 459 patients (18), both demonstrated the significant benefit of adding chemotherapy to RT in terms of progression-free survival, LRC, and OS rates. Notably, the addition of chemotherapy in these trials was associated with a substantial reduction in the risk of locoregional recurrence and demonstrable improvements in disease-free survival. A combined analysis by Bernier et al. (19). further emphasized the significance of ENE and positive margins as critical determinants of unfavorable outcomes. While these investigations did not specifically focus on SGCs, the identified prognostic factors of ENE and positive margins have become widely accepted as crucial indicators for integrating CRT into the management of head and neck cancer. Nevertheless, ongoing debates persist due to the inherent chemoresistance often observed in SGCs, as evidenced by objective response rates ranging from 0% to 44% (20).

The comparison between CRT and RT in terms of prognosis for SGC has attracted extensive research attention. A study by Amini et al. (21) analyzed 2210 SGC patients from the NCDB, revealing compromised OS with adjuvant CRT compared to RT alone. Similarly, Cheraghlou et al. (22) stratified 8580 SGC patients from the same database and observed that the addition of adjuvant therapy conferred an enhanced OS among early-stage disease with adverse features and late-stage disease with adverse features, particularly when involving RT. In contrast, analyses by Gordon et al. (23) and Aro et al. (24) indicated a declining trend in the utilization of CRT and failed to demonstrate a survival advantage with the addition of chemotherapy to RT, despite the inclusion of large NCDB cohorts. Notably, discrepancies in defining high-risk variables were identified, potentially resulting in the oversight of critical prognostic factors such as the extent of involvement, PNI, and LVI. In our study, we meticulously accounted for numerous potential adverse characteristics and concluded that CRT did not confer improvements in OS or LRC, irrespective of PNI status, thereby providing valuable insights into SGC management. It is important to note that while certain studies have suggested longer OS in the CRT group, particularly in cases of salivary gland squamous cell carcinoma (25), caution is advised in the interpretation of these results, as the potential inclusion of cases with metastasis from cutaneous malignancies could impact these findings.

Limitation in current study must be acknowledged, first, there was inherent selective bias within a retrospective study, second, our analysis was based on a single constitution, external validation was required.

In conclusion, the occurrence of PNI was intricately linked with other adverse pathologic features, with both minor and major nerve PNI exerting similar influences on OS and LRC. Furthermore, the addition of chemotherapy to RT did not confer improvements in OS or LRC, regardless of PNI status. This study represents a pioneering effort to comprehensively analyze the distribution of PNI and elucidate its substantial implications for patient survival, thus providing valuable insights for the management of patients in this context.

## Data Availability

The original contributions presented in the study are included in the article/**Supplementary Material**. Further inquiries can be directed to the corresponding author.

## Supplementary material

Supplementary Table S1 Impact of PNI status on overall survival and locoregional control determined by primary sites.
