# Supplementary material for: Major and minor perineural invasion in salivary gland cancer
Source: Front Oncol. 2025 Jan 17;14:1466196. doi: 10.3389/fonc.2024.1466196 (PMC11782203; doi:10.3389/fonc.2024.1466196)
Supplement: Supplementary Table S1 — Impact of PNI status on overall survival and locoregional control determined by primary sites. [file Table1.doc]

Supplementary Table 1. Impact of PNI status on overall survival and locoregional control determined by primary sites.

| Primary site | Overall survival | | Locoregional control | |
| --- | --- | --- | --- | --- |
|  | p | HR [95%CI] | p | HR [95%CI] |
| Parotid |  |  |  |  |
| Minor (n=42) |  | ref |  | ref |
| No (n=238) | <0.001 | 0.56 [0.25-0.87] | <0.001 | 0.67 [0.32-0.88] |
| Major (n=15) | 0.115 | 1.23 [0.75-1.89] | 0.231 | 1.29 [0.71-2.04] |
| Submandibular |  |  |  |  |
| Minor (n=18) |  | ref |  | ref |
| No (n=51) | 0.011 | 0.87 [0.46-0.97] | 0.024 | 0.79 [0.50-0.98] |
| Major (n=8) | 0.437 | 1.47 [0.36-2.05] | 0.621 | 1.66 [0.45-3.06] |
| Sublingual |  |  |  |  |
| Minor (n=10) |  | ref |  | ref |
| No (n=10) | 0.018 | 0.48 [0.18-0.87] | 0.035 | 0.56 [0.32-0.95] |
| Major (n=80) | 0.634 | 1.87 [0.32-3.86] | 0.583 | 1.79 [0.41-4.38] |
| Minor gland |  |  |  |  |
| Minor (n=30) |  | ref |  | ref |
| No (n=110) | <0.001 | 0.79 [0.64-0.95] | <0.001 | 0.84 [0.71-0.97] |
| Major (n=15) | 0.476 | 1.61 [0.64-3.77] | 0.558 | 1.83 [0.73-4.37] |
